# Case Report: Long-term outcomes after surgery for spinal infiltrative lipoma in two dogs

**DOI:** 10.3389/fvets.2026.1778389

**Published:** 2026-08-21

**Authors:** Lucinda L. Van Stee, Sarah J. Van Rijn, Wilhelmina Bergmann, Björn P. Meij

**Affiliations:** Department of Clinical Sciences, Faculty of Veterinary Medicine, Small Animal Surgery, Utrecht University, Utrecht, Netherlands

**Keywords:** dog, infiltrative lipoma, neurosurgery, spinal cord compression, spine, surgical oncology

## Abstract

**Introduction:**

An eight-year-old male intact shepherd mixed breed dog (case 1) and a 6-month-old intact female Picardy sheepdog (case 2) were referred to a Veterinary Teaching Hospital with the diagnosis of infiltrative lipoma causing spinal cord compression.

**Case presentations:**

Case 1 presented with a history of intermittent tremors and right forelimb lameness. Clinical examination revealed an expansive, ill-defined, non-movable, soft tissue mass palpable on the right hemithorax and a mild pelvic limb paraparesis. Computed tomography (CT) revealed a large mass with evidence of infiltration into the thoracic vertebrae and spinal canal at the level of the thoracic (T) vertebrae 1-3. En bloc resection of the extra-spinal portion of the mass was performed followed by careful debridement of the affected spinal processes and removal of the spinal canal portion of the tumor. Histopathological evaluation confirmed an infiltrative lipoma with compressive osteolysis of the dorsal spinal processes. Within 24 hours after surgery, clinical signs disappeared. Follow up CT at 2 and 38 months, and examination at 10, 24, 32 and 38 months after surgery showed no evidence of remaining tumor mass or recurrence of clinical signs. The dog died of non-related disease at 51 months after surgery. Case 2 presented with a large, and slow growing mass over the right lumbar area, that had been present since birth. The dog did not show any clinical or neurological signs. MRI identified a mass extending from T13 to the 6th lumbar (L) vertebrae, and infiltration into the spinal canal with spinal cord compression at the level of Ll-L5. En bloc resection of the extraspinal portion of the mass was performed, followed by meticulous removal of the involved parts of the vertebrae and intraspinal portion of the mass. Histopathological evaluation confirmed infiltrative lipoma, with no obvious infiltration of the bone. Follow up MRI at 6, 18 and 30 months, showed remnant lipoma tissue in the retroperitoneal space without spinal involvement. At 45 months after surgery, the dog was still free of clinical signs.

**Conclusion:**

It is concluded that en bloc resection with meticulous local debridement of infiltrative lipomas causing spinal cord compression can render long-term local disease control.

## 1 Introduction

Infiltrative lipoma is a less common entity in veterinary medicine (1). Its infiltrative behavior and resemblance to normal adipose tissue make it impossible to differentiate between normal and abnormal adipose tissue, both macroscopically and microscopically (1, 2). Recognizing its infiltrative behavior prior to surgical excision is crucial in surgical planning and in achieving complete resection. However, in many cases, large areas will be affected at the time of diagnosis, and curative surgical may no longer be a viable option (3, 4). Infiltrative lipomas generally do not metastasize; however, their local mass effect can compromise locomotion, displace organs, or compress the spinal cord.

Infiltrative lipoma with pain and pelvic limb monoparesis or paraparesis as a presenting complaint is a rare entity (3, 5–13). Curative intent surgery in cases of infiltrative lipoma with spinal canal involvement has been reported in several regions of the vertebral column in the dog (6–12). In the cervical area, the lipoma caused hemiparesis (7). In the thoracic vertebrae, the lipoma caused chronic paraparesis in one case (8) and acute paraparesis in another case (5). In the lumbar vertebral column, the lipoma caused no clinical signs in one case (9) and an abnormal gait of the left pelvic limb in another case (6). Follow-up times of up to 24 months have been reported (5–9). Bone involvement in infiltrative lipoma is extremely rare (7–12), and long-term outcomes after surgery alone have only been reported in one case report (12).

The present case series aimed to describe the presentation, treatment, outcome, and histopathology in two dogs with infiltrative lipoma affecting the spinal canal with radiological and macroscopic evidence of destruction of cortical bone involving several vertebral laminae and dorsal spinous processes.

## 2 Case description

### 2.1 Case 1

A 10-year-old intact male Shepherd mixed-breed dog was referred to the Small Animal Veterinary Teaching Hospital (SA-VTH) with clinical signs of intermittent right forelimb lameness for 2-3 months prior to presentation, intermittent tremors of the right forelimb when recumbent, and a growing mass on the right thoracic wall.

General clinical examination showed asymmetry of the dorsal thoracic/suprascapular region with a marked mass effect on the right dorsal hemithorax. Gait observation revealed very mild pelvic limb paraparesis. Neurological examination was unremarkable except for the aforementioned paraparesis and a pain response on deep palpation of the area under the mass. The mass had been recently biopsied; thus, post-surgical pain could not be ruled out. Complete blood cell count and serum biochemistry were unremarkable.

**Figure 1:**
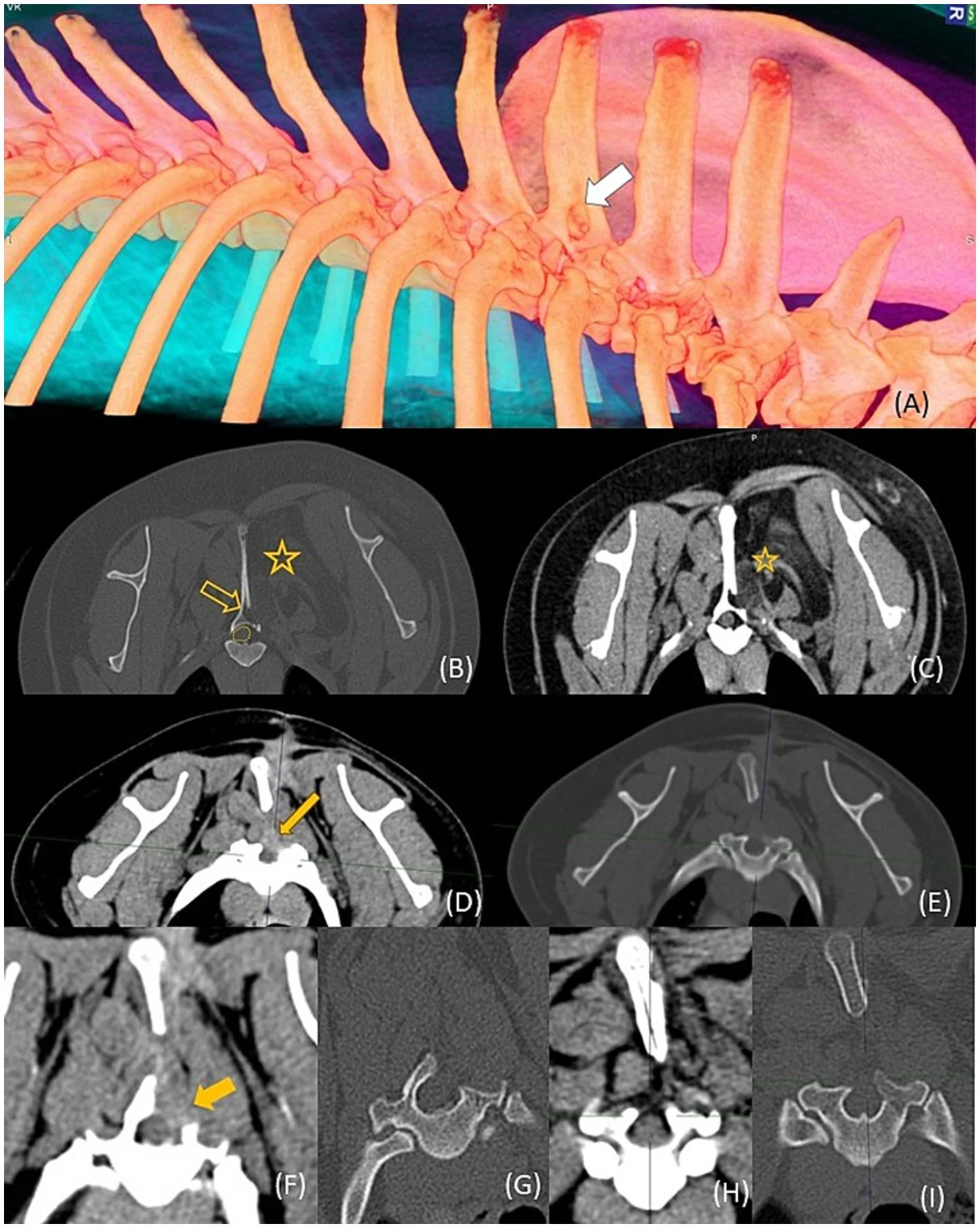
Case 1 (Ten-year-old male intact shepherd mixed breed dog presented with intermittent right front limb lameness). **(A)** 3D reconstruction demonstrating the osteolytic lesion at the base of spinous process of the T3 vertebra (white arrow). **(B)** CT transverse reconstruction of the presurgical computed tomography in bone setting at the level of T3 vertebrae. An osteolytic lesion (arrow) at the base of T3 spinous process, with left lateral displacement of spinal cord (yellow dotted line) due to lipoma (star) invasion into the spinal canal. **(C)** Preoperative CT, transverse reconstruction of the presurgical computed tomography in soft tissue setting. At the level of T3, marked spinal compression is evident: the intraspinal hypodense fatty mass can be clearly distinguished from the compressed spinal cord that is isodense to the surrounding spinal muscles. **(D)** Transverse reconstruction (soft tissue setting) taken from the 3 month postoperative CT scan, showing previous hemilaminectomy site at T2 with no signs of recurrence of adipose tissue at the level of the previous surgery.**(E)** Transverse reconstruction of the 3 month postoperative CT scan at the level of T2 in bone setting at the same level of image 1D. **(F)** Transverse reconstruction in soft tissue setting of the 10 month post-operative CT scan, showing previous hemilaminectomy site at T2 with no signs of recurrence of adipose tissue at the level of the previous surgery. **(G)** Transverse reconstruction (bone setting) of the same location of image 1F. **(H)** Transverse reconstruction in soft tissue setting of the 38 month post-operative CT scan, showing previous hemilaminectomy site at T3 with no signs of recurrence of adipose tissue at the level of the previous surgery. **(I)**. Transverse reconstruction in bone setting of the same location of image 1 H.

Computed tomography (CT) of the thoracic spine revealed a mass with an extraspinal component (90%) and an intraspinal component (10%) located at the right sagittal axis of the spinal column. Both the extraspinal and intraspinal mass components had an adipose tissue attenuation of −170 (±5) Hounsfield Units (HU) on CT with minimal contrast enhancement. The extraspinal component measured 17 cm × 6.5 cm × 5.5 cm and extended across the thoracic (T)1 to T8 vertebrae, creating a mass effect, pushing epaxial musculature laterally, and showing an infiltrative behavior within the epaxial muscles, as well as a compressive effect on the surrounding musculature. Between T1 and T3, the tumor invaded the spinal canal through the T1-T2 and T2-T3 intervertebral foramina on the right side and also the base of the dorsal spinous processes of T2 and T3 through well-defined osteolytic punch-hole-like lesions (Figure 1A). The spinal canal was filled with the intraspinal tumor mass component measuring 3 cm × 0.5 cm × 0.5 cm, pushing the spinal cord laterally (Figures 1B,C). CT showed no signs of pulmonary or regional metastasis. Fine-needle aspiration biopsy confirmed the presumptive diagnosis of lipoma.

The dog was premedicated using fentanyl (3 μg/kg *intravenously* [IV]) and ketamine (0.4 mg/kg IV). Anesthesia was induced with propofol on effect (3.4 mg/kg IV) and was maintained with isoflurane inhalant. Perioperative and postoperative systemic analgesia included constant-rate infusions (CRIs) of ketamine (5 μg/kg/min), fentanyl (10 μg/kg/h), and lidocaine (50 μg/kg/min). Local analgesia included chirocaine (0.7 mg/kg) as a local splash block. Perioperative antibiotic prophylaxis consisted of cephazoline (20 mg/kg IV) and was repeated every 90 min. Monitoring included electrocardiography, capnography, and measurement of oxygen saturation and non-invasive arterial blood pressure.

**Figure 2:**
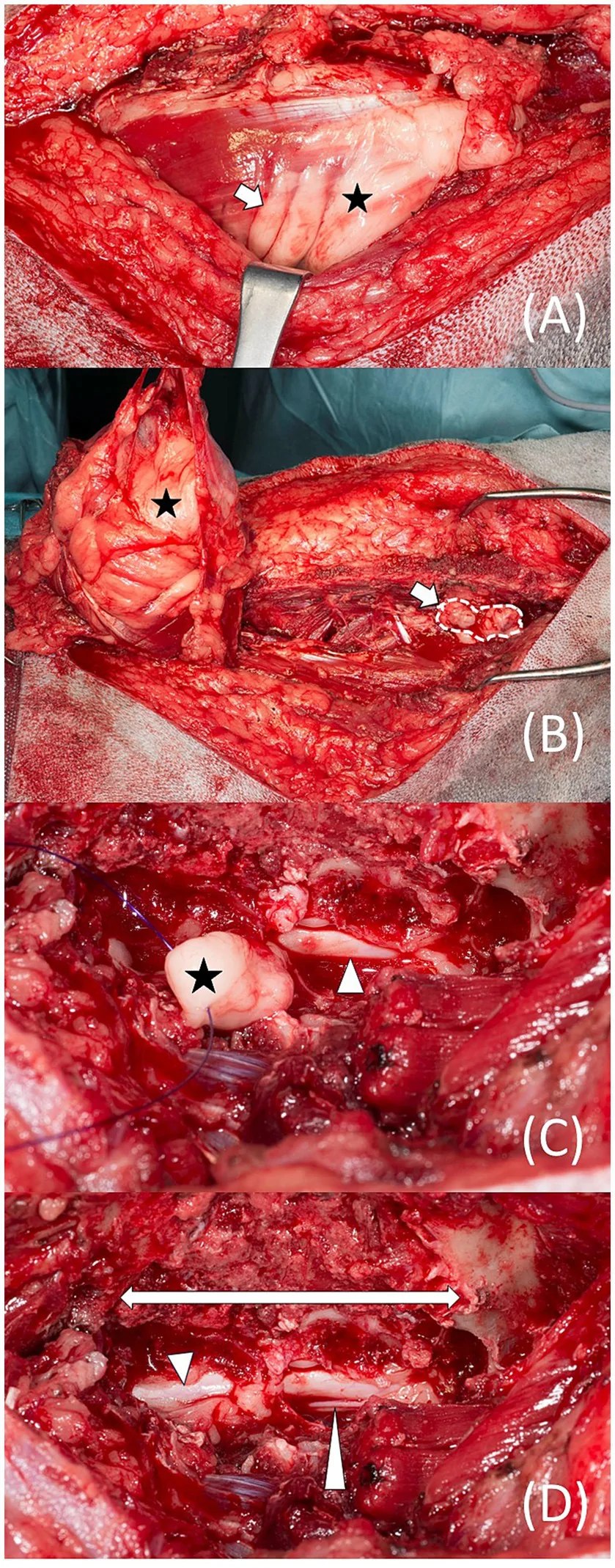
Case 1 (10-year-old intact male Shepherd mixed-breed dog presented with intermittent right front limb lameness). **(A)** Intraoperative image showing the infiltrative nature of the lipoma (star), invading into the muscle longissimus thoracis (arrow). **(B)** After en bloc resection of the tumor (star) affecting the muscle longissimus thoracis, the intraspinal portion protrudes (arrow) from the osseous defect in the spinal column. **(C)** A stay suture is placed through a small part of the remainder of the mass (star) that is still present in the spinal canal. This portion of the mass is then carefully lifted off the spinal cord (arrowhead). Within the spinal canal, this part of the mass appears to be well-defined and not obviously infiltrative in nature onto the dura. **(D)** After removal of the intraspinal portion of the tumor, the affected bone overlying the spinal cord is further debrided, leaving a large defect in the vertebral arch (double-headed arrow). The spinal cord (thick arrowhead) and nerves (thin arrowhead) appear normal and not infiltrated by the tumor.

The dog was placed in a slightly slanted left lateral recumbent position for surgery. The mass was resected in two stages. First, the extraspinal portion was addressed, followed by resection of the intraspinal portion of the mass. Surgical loupes were used for intraoperative magnification. Hemostasis was achieved using monopolar and bipolar electrocautery. A dorsal skin incision at the level of the dorsal spinous processes was made, extending from C6 to T10. After skin incision, the right-sided epaxial musculature was protruding through the subcutaneous tissues, immediately revealing the invasion of the musculature by the underlying infiltrative lipoma (Figure 2A). The epaxial musculature was detached from its origin at the dorsal spinous processes using monopolar electrocautery and retracted laterally, exposing the bulk of the tumor mass located on the ventral aspect of the epaxial musculature (Figure 2B). A small finger-like adipose tissue strand was located at the base of the epaxial musculature at T2 and T3. The extraspinal portion of the tumor was removed, including all invaded musculature, from the cranial margin at T1 down to the caudal edge of the lipoma at the level of T8, after which the caudal two-thirds of the wound was closed. Musculature was closed using interrupted sutures (2-0 polydioxanone); the midline thoracic fascia was closed using a simple continuous suture pattern (2-0 polydioxanone). Subcutaneous tissues were closed using a simple continuous suture pattern (2-0 poliglecaprone 25), and the skin was closed using a subdermal continuous suture pattern (3-0 poliglecaprone 25). The remaining wound exposed the C7-T4 vertebral area, and the dorsal spinous processes of T1, T2, and T3 were freed from their soft tissue attachments. The infiltrative lipoma affected the base of T2 and T3 dorsal spinous processes, which were then removed using a 4 mm high-speed electric burr and Kerrison rongeurs. The supraspinous ligament and the proximal half of the spinous processes of T1-T3 were left *in situ*. A dorsal laminectomy was performed, extending from the caudal one-third of T1, over the full length of T2 and T3. At the level of T2 dorsal laminectomy, a 2 × 1 cm rounded adipose tissue mass was present within the spinal canal. This mass had well-defined margins and was, macroscopically, not infiltrating the neural tissues or bone within the spinal canal. The mass was lighter and denser in structure compared to the surrounding epidural fat. The mass was carefully elevated from the spinal canal, revealing the underlying spinal cord and nerve roots. Immediately after tumor removal, the impressed spinal cord started to return to its original round shape (Figure 2C). The spinal cord continued to swell, and mannitol (0.5 g/kg IV) and dexamethasone (0.2 mg/kg IV) were administered to counteract spinal cord edema.

The cranial part of the wound was closed in the same manner as described for the caudal part of the wound. Amoxicillin–clavulanate (IV 20 mg/kg every 8/12 h [TID / BID]) was administered throughout the hospitalization period. Twelve hours after surgery, the dog showed no obvious lameness. He was discharged 48 h after surgery with the following medications: carprofen (2 mg/kg *per os* [PO] for 14 days, BID), tramadol (4 mg/kg PO for 10 days, TID), amoxicillin–clavulanate (12.5 mg/kg PO for 10 days, BID), and ranitidine (2 mg/kg PO for 14 days, BID). A progressively increasing exercise schedule was implemented for the first 6 weeks postoperatively, consisting of incremental steps of 5 min/week of leash walking, 3–5 times per day. The dog was evaluated by a veterinary physiotherapist at 10 days postoperatively, who concluded that no further physiotherapy or hydrotherapy was necessary.

Histopathological evaluation of the excised tissue was consistent with an infiltrative lipoma. The excised parts of the spinous processes of T2 and T3 showed cortical and trabecular bone with bone marrow, surrounded by well-differentiated muscle and adipose tissue. Locally, multifocal indentation of cortical bone structure was noted. Focally, a large cavitation filled with well-differentiated adipose tissue was noted, and the interface between the adipocytes and trabecular and cortical bone was marked by a well-defined lining of osteoblasts (Figure 2D). Within the adipose tissue in this cavitation, small hemorrhagic areas with mild fibroblast proliferation were present. A few bone marrow-derived cells were present in the periphery of this process.

Twelve weeks after surgery, the dog returned for clinical examination and repeat CT (Figures 1D,E). No changes were found during orthopedic and neurological examination compared to the first visit. CT of the thoracic spine revealed tiny areas of fat, interpreted as normal pre-existent fat tissue. The right dorsal lamina and ventral part of the spinous processes of T1, T2, and T3 were absent as expected after surgery. At this level, soft tissue attenuation was found in direct contact with the spinal cord, with no signs of local recurrence of the infiltrative lipoma or spinal cord compression or deformation. A small amount of irregular but well-defined periosteal new bone formation was noted along the right side of the spinous processes of T2 and T3. No evidence of remnants of the infiltrative lipoma or spinal cord compression was noted at the level of the previous hemilaminectomy sites (Figures 1D,E).

At 10 months post-surgery, CT was repeated at the referring veterinary teaching hospital near the owners’ hometown, with a stable presentation (Figures 1F,G). At 24, 32, and 38 months after surgery, follow-up examination of the dog showed no further changes in the orthopedic and neurological status. At 38 months post-surgery, repeat CT imaging (Figures 1H,I) showed a stable presentation. At the age of 14 years and 5 months (51 months after surgery), the dog was euthanized due to severe refractory chronic pancreatitis. At this time, no changes were observed in orthopedic and neurological status.

### 2.2 Case 2

A 6-month-old female Picardy sheepdog was referred to the SA-VTH with a subcutaneous lumbar mass that had been present since birth. The mass had been growing steadily over time and extended over a large part of the right lumbar area. No other clinical signs were found, and general physical examination and neurological examination were unremarkable. Fine-needle aspirate cytology samples of the large mass were consistent with adipose tissue.

One month after the initial presentation, magnetic resonance imaging (MRI) of the lumbar spinal column was performed. MRI identified a large subcutaneous T1-weighted (T1W) and T2-weighted (T2W) (Figures 4A,B) hyperintense mass on the right dorsal side of the lumbar vertebral column (Figures 4A,B). The mass infiltrated the musculature on the right side from the level of T13–L6, with almost complete involvement of the multifidus and longissimus lumborum muscles at the level of L2–L5. The mass extended along the lateral and ventral aspects of the iliocostalis lumborum muscle. At the level of L4, the mass infiltrated the abdominal cavity. The dorsal spinous processes of L3 and L4 were completely enveloped by the mass. The intervertebral foramina of L2-L3 and L3-L4 were enlarged, and an increased amount of fat was visible inside the spinal canal at the level of L1–L5 with displacement of the spinal cord to the left. The right transverse processes of L3-L4 showed osseous proliferation with contrast enhancement. Mild asymmetry of the L2–L5 vertebrae was noted.

**Figure 3:**
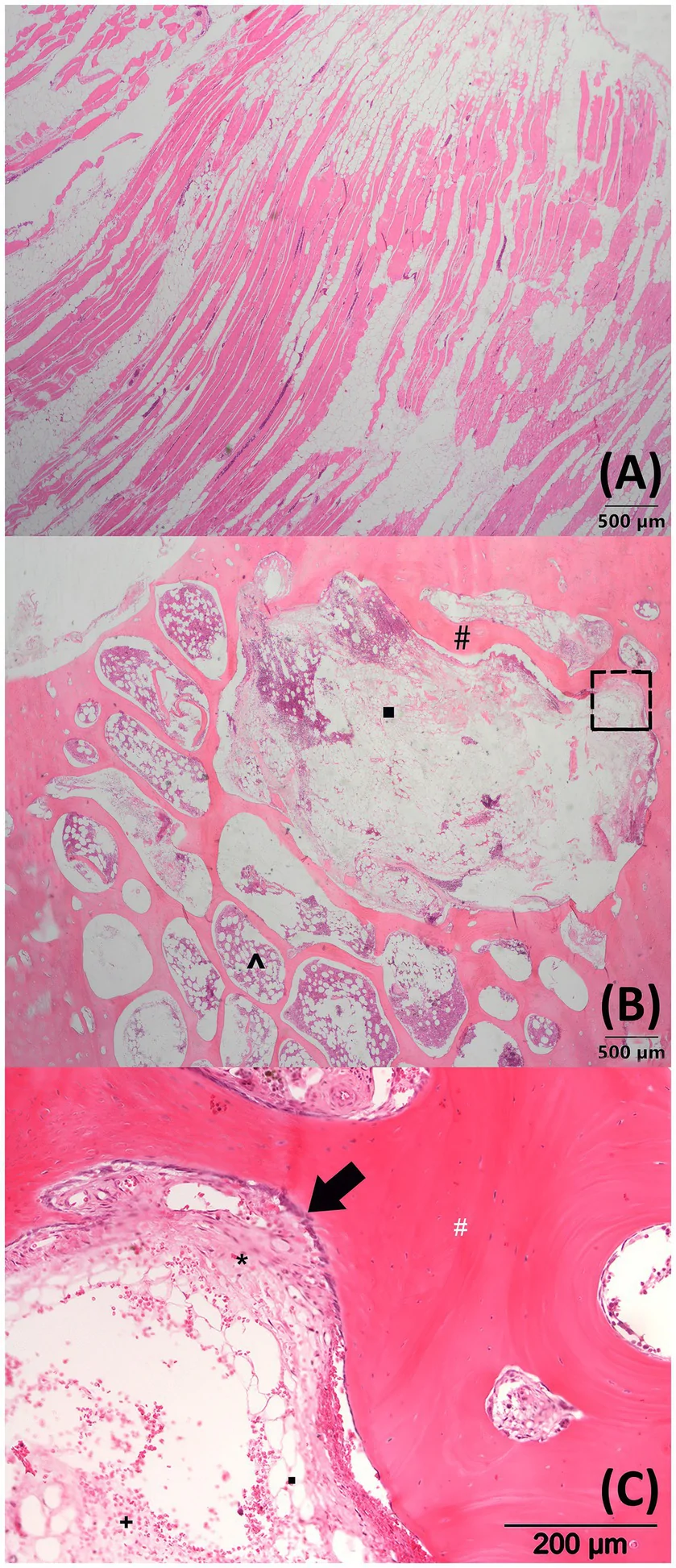
Histology plate case 1 (10-year-old intact male shepherd mixed-breed dog presented with intermittent right front limb lameness). **(A)** Photomicrography of a muscle infiltrated by the infiltrative lipoma. **(B)** Overview photomicrography of the spinal process with the infiltrative lipoma. (■), (<) bone marrow; trabecular bone (#). The rectangle indicates the area of the lipoma enlarged in this figure. **(C)** Photomicrography of the interface between the infiltrative lipoma and the vertebral bone. Within the lipoma (■) are small hemorrhagic areas (+) and fibroblast proliferation (*). The arrow points to osteoblasts lining the trabecular bone.

The dog was premedicated and anesthetized with the same protocol as the first case. The perioperative antibiotic prophylaxis protocol and intraoperative monitoring were identical to case 1.

The dog was placed in sternal recumbency for surgery. Surgical loupes were used for intraoperative magnification. Hemostasis was achieved using bipolar electrocautery. A right paramedian skin incision was made from T13 to S1. The subcutaneous tissues were sharply dissected, and the mass was circumferentially mobilized using a combination of sharp and blunt dissection on the caudal, left, and right aspects down to the underlying fascia. A stab incision in the dorsal fascia was made at the midline just cranial to the dorsal spinous process of L5. Dissection was extended left paramedian in a cranial direction beyond the cranial margin of the mass. It was then returned to the midline and extended to the right. The left multifidus musculature was dissected from L2 to L5 to expose the laminae. The dorsal spinous processes of L3 and L4 were removed using a high-speed burr. The hyperostotic L3-L4 facet joint was removed using a combination of high-speed burring and rongeurs. The mass demonstrated infiltrative growth and was firmly adherent to the musculature of the right abdominal wall and was excised en bloc, during which the abdominal cavity was inadvertently entered at the level of the right kidney. Residual mass located ventral to the transverse processes of L3-L4 was carefully dissected free and excised. A right-sided hemilaminectomy was performed at L2–L5 using a high-speed burr. The mass was carefully and bluntly dissected from the neural tissue in a caudal-to-cranial direction using a nerve hook. The right L3 spinal nerve root was found to course entirely through the mass and continue ventrally beneath the transverse process, corresponding to the previously excised infiltrative component. Due to complete involvement, the nerve root was sharply transected and resected.

The abdominal wall was closed using simple cruciate sutures (0 polydioxanone). The dorsal fascia was closed primarily with mattress sutures (0 polydioxanone). Multiple tacking sutures (0 polydioxanone) were placed to eliminate dead space. A Jackson–Pratt drain was placed with a right craniodorsal exit site and secured to the skin using a Chinese finger-trap suture (3-0 nylon). The subcutaneous tissue was closed in two layers using simple interrupted and continuous sutures (3-0 poliglecaprone 25). The skin was closed using simple cruciate sutures (3-0 nylon).

Gait assessment at 12 and 36 h after surgery showed mild pelvic limb paresis, which improved over time. There was a decreasing amount of fluid production in the Jackson–Pratt drain, which was removed before discharge. The dog was discharged 48 h after surgery, and the following medications were prescribed: carprofen (2 mg/kg PO for 14 days, BID), gabapentin (10 mg/kg PO for 21 days, TID), and amoxicillin–clavulanate (12.5 mg/kg PO for 5 days, BID). A progressively increasing exercise schedule was implemented as in case 1.

Histopathological evaluation of the excised tissue was consistent with an infiltrative lipoma within the soft tissues of the intraspinal and extraspinal sections that were analyzed. No clear infiltration into the bone was observed.

Two weeks after surgery, the dog returned for clinical examination and removal of the skin sutures. Mild seroma formation was observed on the ventral side of the left lumbar area. The dog’s gait was normal, without paresis or ataxia. At 6 weeks, the seroma had resolved, and physiotherapy was started to improve muscle strength.

**Figure 4:**
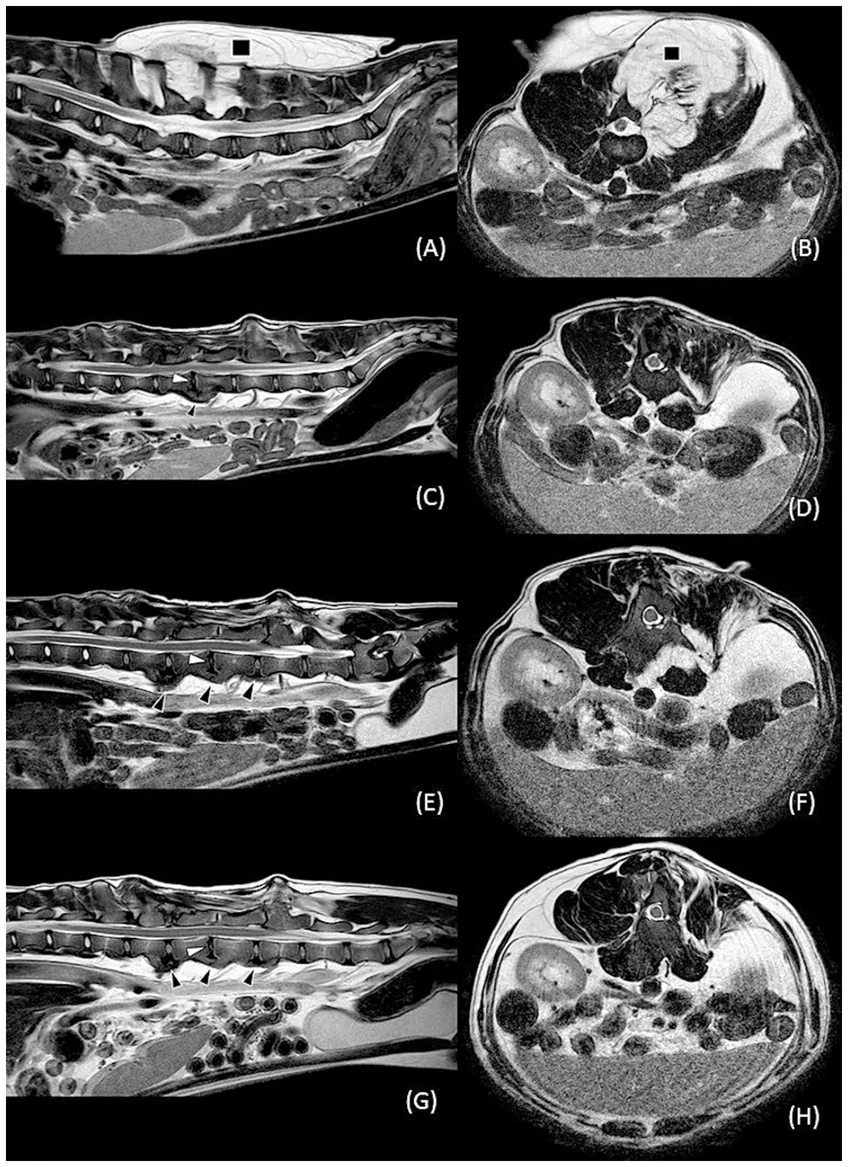
Case 2 (6-month-old female Picardian shepherd with a subcutaneous lumbar mass). **(A)** Preoperative MRI T2-weighted images, sagittal view, and **(B)** transverse view at the level of the L2-L3 intervertebral foramen. Hyperintense lipoma mass (■) on the right dorsal side of the lumbar vertebral column. The mass infiltrated the musculature on the right side from the level of T13–L6, with almost complete involvement of the multifidus and longissimus lumborum muscles at the level of L2–L5. The mass persisted in the lateral and ventral sides of the iliocostalis lumborum muscle. At the level of L4, the mass seemed to infiltrate the abdominal cavity. The dorsal spinous processes of L3 and L4 were completely enveloped by the mass. The intervertebral foramina of L2-L3 and L3-L4 were enlarged, and an increased amount of fat was visible inside the spinal canal at the level of L1–L5 with displacement of the spinal cord to the left. First follow-up at 6 months: postoperative **(C)** MRI T2-weighted images, sagittal view, and **(D)** transverse view at the level of the L2-L3 intervertebral foramen; follow-up at 18 months: **(E)** MRI T2-weighted images, sagittal view, and **(F)** transverse view at the level of the L2-L3 intervertebral foramen; and follow-up at 30 months: **(G)** MRI T2 weighted images, sagittal view, and **(H)** transverse view at the level of the L2-L3 intervertebral foramen. No evident spinal cord compression by the fatty tissue was observed. Furthermore, there were degenerative changes in the intervertebral disk and end plates of L3-L4 initially (white arrowheads), with collapse of the intervertebral space and slight kyphosis with fusion of the dorsal laminae, and progressive ventral spondylosis at L2-L3, L3-L4, and L4-L5 (black arrowheads) and generalized mild intervertebral disk degeneration of the remaining lumbar spine (Pfirrmann grades 2-3).

A follow-up MRI was performed at 6 months (Figures 4C,D), 18 months (Figure 4E,F), and 30 months (Figures 4G,H) postoperatively and showed the presence of right-sided hypaxial lipoma tissue, mildly extending into the retroperitoneal space. This tissue remained stable over the follow-up period.

On orthopedic and neurological evaluation, the dog continued to show mild discomfort on deep palpation of the lumbar spine, but no other abnormalities were observed. The dog was still alive and healthy 4.5 years after surgery.

## 3 Discussion

Two cases of infiltrative lipoma are reported, affecting segments of the spinal column, causing marked local vertebral bone destruction and spinal cord compression, resulting in spinal pain and mild paraparesis of the pelvic limbs in the first case and no obvious clinical signs in the second case.

Although the dog in case 2 did not show neurological signs, the mass effect of the tumor and the expectation that neurological deterioration was inevitable over time led to the decision to proceed with surgical excision. Due to the young age of the dog and to the progressive increase in the size of the mass over time, postponing surgery to wait for clinical signs would likely have increased the extent of the surgical excision. Considering the significant compression of the spinal cord observed on imaging, surgical intervention was deemed inevitable by the surgeons involved.

In both cases, surgical excision was successful and led to long-term remission of clinical signs. The masses were resected in two stages. First, the extraspinal portion of the tumor mass was removed en bloc with affected musculature. In the second stage of the surgery, a careful intralesional approach was chosen.

To gain access to the spinal canal, dorsal laminectomy was performed in case 1 and hemilaminectomy was performed in case 2. The extent of both surgical procedures was considered safe and should not have caused instability of the spinal column. However, this was not specifically assessed during surgery. The dog in case 2 did show progression of spondylosis on follow-up imaging, which could be due to possible spinal instability at the surgery site. However, no clinical signs were noted on follow-up appointments, and a conservative approach of careful monitoring remains in effect.

Spinal instability after spinal decompression surgery is a genuine concern that may affect patient outcomes. The surgical technique used in case 1, leaving as much of the dorsal spinal processes and supporting contralateral paraspinal muscles intact, has been successfully applied in other cases by the authors and is supported by a previous publication (14). By leaving as much of the soft tissue envelope and attachments of paraspinal muscles and ligaments *in situ*, spinal column stability can be maintained (14). However, it has been reported that even small attenuations of the spinal column can lead to clinical deterioration (15). Biomechanical studies on spinal column stability after consecutive and bilateral hemilaminectomy in the thoracolumbar spine showed no reduction in stiffness during flexion and extension in one study (16) and noted the importance of the integrity of the intervertebral disk and facet joints in another study (17). A different study investigated spinal instability after spinal fractures in dog cadavers. The investigators modeled their experimental setup on the three-column concept of spinal stability proposed in 1983 in human medicine (18). The ventral column includes the ventral longitudinal ligament, the ventral two-thirds of the vertebral body, and the ventral two-thirds of the intervertebral disk. The middle column includes the dorsal one-third of the vertebral body, the dorsal one-third of the intervertebral disk, the dorsal longitudinal ligament, the pedicles, the zygapophyseal joints, the flavum ligament, the vertebral arch, and the interconnecting ligament. Spinal instability occurs only when two or all three of the three columns are affected (18). However, if intraoperative or postoperative instability is noted or expected, the authors recommend proceeding with spinal stabilization.

Due to the local anatomy, en bloc resection was not possible for the second portion of the mass without the risk of permanent damage to neural tissue and the resultant loss of neurological function. The aim of the second stage of surgery was to remove the compressive mass within the spinal canal. When performing partial intralesional surgery, meticulous local resection, including curettage of affected bone and piecemeal resection with minimal tumor seeding, is essential to prevent the risk of recurrence (10).

In case 1, intraoperative spinal cord swelling was noted during surgery and was treated with both mannitol and dexamethasone. The 2022 ACVIM consensus statement on acute thoracolumbar intervertebral disk extrusion (IVDE) advises against the use of dexamethasone explicitly because of the risk of gastrointestinal complications and its minimal effect on clinical outcome (19). However, in cases of suspected spinal cord swelling due to edema and possible ischemia/reperfusion and subsequent inflammation caused by intraoperative manipulation (i.e., surgical trauma) or rapid decompression, dexamethasone can be applied in selected cases, although dosage may be lower than that used in case 1 (20, 21). The use of mannitol for decreasing edema in the central nervous system is less controversial; however, adequate fluid therapy is required after surgery, as mannitol is an osmotic diuretic that will cause dose-dependent dehydration (22). Although durotomy has been advocated in the past to alleviate localized spinal cord swelling in cases of acute spinal trauma, the authors of a recent randomized controlled trial in dogs advise against this practice (23).

Histopathological evaluation confirmed the infiltrative nature of the well-differentiated adipose tissue within the tumors. The osteolytic effects on the vertebral bone architecture showed a pattern more consistent with compression, as also suggested in a previous publication (10). The well-defined interface between the adipose tissue and the bone, marked by the presence of an osteoblast lining of the lacunae (Figure 3C), showed no infiltration other than well-defined adipose tissue within the cavity formed by bone lysis that occurred due to compression. The adipose tissue did not infiltrate the bone tissue itself, as shown in Figures 3B,C. The well-defined margins of the tumor–bone interface and the lack of reaction of the bone suggest a relatively slow process, which is more consistent with compressive bone changes (24, 25).

While the tumor infiltrated the soft tissues, both macroscopic and microscopic examinations revealed that the submitted bone harvested from the affected spinous processes showed evidence of compression but no evidence of invasion. These findings show that the masses can have multiple effects on surrounding tissues and are not always infiltrative in all directions. At the level of soft tissues, the mass infiltrated between healthy tissues, thereby changing its architecture (Figure 3A). At the level of the bone, no evidence of infiltration was noted on histopathological assessment; instead, the lesion exhibited a compressive behavior (Figure 3B,C). This heterogeneous behavior of infiltrative lipomas in these cases aligns with the experience of the authors’ clinical experience with infiltrative lipomas in other anatomical locations. As shown in the intraoperative photographs of case 1, the infiltrative lipoma also has well-encapsulated areas on the lateral side of the mass, while finger-like strands of adipose tissue macroscopically invade the underlying muscles (Figures 2A, 3A). Microscopically, no differentiation could be made between the adipose tissue of the infiltrative lipoma and the pre-existent adipose tissue, as both consisted of well-differentiated adipose tissue. This finding is consistent with previous reports and may be the main reason for local tumor recurrence (5–13). There are not many reports in the veterinary literature describing spinal canal infiltration of lipomas, but these encompass a wide range of locations and clinical presentations (5–13). In addition, the two cases presented in this study differed considerably with respect to age, breed, and tumor location.

Local resection of infiltrative lipomas causing spinal compression has been reported previously in eight cases (5–13); however, only in two cases was destruction of vertebrae noted, and in only one case report (10), histopathological evaluation described the bone–tumor interface. Our histopathological findings are consistent with those reported by Raimondi et al. (10). As observed in our cases, long-term local disease control can be achieved by meticulous local resection (10), although differentiation between normal and abnormal fat tissue is difficult, hampering the macroscopic assessment of resection completeness. In the early 2000s, radiotherapy (RT) was reported to be an effective method of treating infiltrative lipomas in dogs, with stable disease achieved in 12 of 13 dogs in one case series (4). More recent retrospective studies evaluating the efficacy of RT in infiltrative lipomas have underlined its value, as both consisted of postoperative RT and as a primary therapy (26–28). The tumor in the two cases presented in this study invaded the spinal canal, and the postoperative radiation field would have included the thoracic or lumbar spinal canal over the length of several vertebrae, resulting in the spinal cord being exposed to a relatively extensive dose of radiation. RT as either a primary treatment or postoperative adjuvant therapy was considered but ultimately rejected as a viable option due to the risk of radiation-induced myelopathy.

## 4 Conclusion

The present cases demonstrate the locally very aggressive nature of infiltrative lipomas invading the spinal canal, leading to compressive bone destruction. When en bloc resection is no longer feasible due to the proximity of the spinal cord, meticulous resection of the spinal compressive portion of the infiltrative lipoma may result in a favorable long-term outcome. Because this study describes only two cases, these findings should be interpreted with caution and should not be generalized to infiltrative lipomas at other anatomical locations.

## Data Availability

The raw data supporting the conclusions of this article will be made available by the authors, without undue reservation.
